# Psychosocial Support Programs for the Mental Well-Being of High School Learners From Low- to Middle-Income Countries: Protocol for a Scoping Review

**DOI:** 10.2196/78035

**Published:** 2025-12-30

**Authors:** Esther Caroline Malo, Doreen Asentewa Abeasi, Elizabeth Sipiwe Ndofirepi, Nokuthula Gloria Nkosi

**Affiliations:** 1 Gauteng College of Nursing, No. 37 Plunkett Avenue, Hursthill, Johannesburg, Gauteng, 2092, South Africa, 27 11644 8942; 2 Nursing and Midwifery, Health Science, Presbyterian University, Agogo, Ghana; 3 Student Success, Health Sciences, University of the Witwatersrand, Johannesburg, South Africa; 4 Nursing Education, Health Sciences, University of Witwatersrand, Johannesburg, South Africa

**Keywords:** psychosocial support programs, mental well-being, high school, learners, low- to middle-income countries, LMIC

## Abstract

**Background:**

The World Health Organization reported in 2020 that approximately 50% of all mental health disorders in adolescents manifest before the age of 14 years. However, the literature on mental well-being and programs designed and implemented by nurses for adolescents in low- to middle-income countries (LMICs) is limited. This scoping review explores the development and implementation of psychosocial support programs targeting high school learners in LMICs.

**Objective:**

This prospective scoping review will examine the psychosocial support programs that exist and how they have been implemented for high school learners from LMICs.

**Methods:**

Using the Joanna Briggs Institute scoping review framework, we will identify primary research articles through systematic searches of the ERIC, MEDLINE, ScienceDirect, PubMed, and PsycInfo databases. Gray literature will also be sourced from Google Scholar. Two independent reviewers will apply the predetermined inclusion criteria to select studies. Data will be charted, analyzed narratively, and presented in tables and figures.

**Results:**

This review will analyze the psychosocial support programs for high school learners in LMIC, identifying gaps in the literature and highlighting areas for further investigation, thereby aiding in creating or adjusting such programs. This study is not funded, and data collection was completed in May 2025. Data analysis is currently in progress, and the results are expected to be finalized and submitted for publication in February 2026.

**Conclusions:**

This scoping review will synthesize evidence on psychosocial support programs in LMICs and guide the development of targeted interventions to address the mental health needs of high school learners.

## Introduction

The World Health Organization reported in 2020 that approximately 50% of all mental health disorders in adolescents manifest before reaching the age of 14 years globally. For instance, 1 in every 5 adolescents receives a diagnosis of a mental disorder, with three-quarters of them experiencing the onset of initial symptoms before reaching 24 years of age [1]. In China, 41.8% of high school students reported mental health problems, with academic stress being the most common issue [2]. A meta-analysis of Chinese studies found depression (28%) and anxiety (26%) to be the most prevalent problems [3]. Similarly, in Jordan, West Asia, anxiety and depression were associated with higher suicide risk among high school students [4]. Comparatively, in Africa, high school students in Ghana experienced more substantial mental health challenges than university students, including higher levels of academic stress, depression, and suicidal ideation [5]. This highlights the need for more psychosocial support for high school learners.

Academic concerns have been identified as major contributors to mental health deterioration [6,7] among high school learners, with academic stress being reported as the most frequent mental health problem [8]. Other notable factors include sleep pattern disruption, the extended use of electronic devices, and social isolation [6,7]. Pre-existing conditions, family dynamics, and the school environment also play crucial roles [8,9]. The interaction of individual, family, school, and social factors markedly shapes adolescent mental health [9].

It has been reported that students with mental health challenges feel they may be unable to pursue their studies [10,11]. Additionally, they may not fully integrate with their peers and social circles, which could negatively affect their interpersonal communication skills. Studies have identified that those who deal with mental health challenges at the adolescent stage are more likely to experience mental health challenges when they enter adulthood [12,13]. The authors add that psychological disorders such as eating disorders, self-harm, and suicide have been reported late in adulthood.

Mental health interventions for adolescents in school settings show promise but require more robust evidence. School-based programs using cognitive behavioral therapy, life skills, and coping skills curricula have demonstrated reductions in depressive symptoms and improvements in problem-solving and overall well-being [12,13]. However, most of these studies have small sample sizes, tend to heavily rely on self-report measures, and are mostly conducted in high-income countries, and as such, their applicability and generalizability in LMICs are questionable.

Although various mental health interventions exist for adolescents in sub-Saharan Africa and LMICs, they are limited in geographic availability and often lack detailed reporting on their development and implementation [14]. Additionally, although some studies examine adolescent mental health in general, there is insufficient research specifically on interventions designed for high school learners in LMICs [15]. High schools are a critical setting for psychosocial support because they provide direct and sustained access to adolescents during a period of heightened vulnerability. Therefore, a scoping review is needed to bring together what is already known about psychosocial support programs in high schools in LMICs, showing what has been tried, what is missing, and what works in specific contexts. The findings will help guide the design of a tailored psychosocial support program that fits the realities of LMIC settings.

## Methods

### Overview

The Joanna Briggs Institute (JBI) framework proposed by Peters et al [16,17] will be used for this scoping review. The PRISMA-ScR (Preferred Reporting Items for Systematic Reviews and Meta-Analyses Extension for Scoping Reviews) flowchart will be included in the final review to illustrate the identification, screening, and inclusion of studies (Checklist 1). The steps are explained in subsequent sections.

### Aligning the Objectives and Research Questions

This prospective scoping review will examine how psychosocial support programs have been developed and implemented for high school learners from LMICs.

The research question for the scoping review is “How have psychosocial support programs been developed and implemented for high school learners from LMICs?”

### Making the Inclusion Criteria Relevant to the Objectives and Questions at Hand

The inclusion criteria will be based on the population, concept, and context (PCC) format to maintain consistency with the objectives and research topic [17]. Textbox 1 shows a clear relationship between the research question and objectives, which also specifies the PCC. The objectives describe what is expected for the scoping review. Each objective should directly address the research question, which is vital.

Textbox 1. **The relationship between the research question and objectives.**

**Objective**

- The objective is to describe psychosocial support programs developed and implemented for learners in high schools in low- to middle-income countries between 2013 and 2023.

**Research question**

- How have the psychological support programs been developed and implemented for high school learners from low- to middle-income countries?

**Population**

- High school learners
- Methodological papers that did not include a population but described a tool, its development, and psychometric properties.
- “High school learners” AND/OR “pupils”

**Concept**

- Psychosocial support programs that promote the mental well-being of high school learners
- “Psychosocial support programmes” AND/OR “strategies”

**Context**

- Low- to middle-income countries

### Explaining the Intended Strategy for Finding, Identifying, Retrieving, Interpreting, and Presenting Evidence

This scoping review will follow the 3-step technique provided by the JBI framework, since scoping reviews typically take a comprehensive approach using both qualitative and quantitative articles. Peer-reviewed and gray literature will also be included.

### Conducting the Search for Evidence

#### Initial Search

A restricted search of at least 2 databases that apply to the study will be conducted in the initial stage. The objective of this first search is to analyze text terms in the publications that were found, paying particular attention to the abstract and title. The intended strategy for this scoping review includes a primary search that will be conducted using the terms “mental well-being,” “psychosocial support programme,” “high school,” and “learners” on 2 electronic databases, such as PubMed and PsycInfo. The search terms will be connected using Boolean operators like “OR” and “AND.”

#### Secondary Search

Databases to be searched include ERIC, MEDLINE, ScienceDirect, PubMed, and PsycInfo. Keywords such as “psychosocial support programmes,” “mental well-being,” and “high school learners” and their synonyms will be used with Boolean operators. The search will be supplemented by reviewing references of selected articles.

The researcher and a librarian from the Faculty of Health and Medical Sciences library at the University of Witwatersrand will work together to establish the final search strategy.

Examples of keywords that will be used in the ERIC database search are “psychosocial support programmes” OR “psychosocial intervention” OR “psychoeducation” OR “emotional support” OR “nursing therapy” OR “psychosocial support” OR “mental health programme” AND “mental well-being” OR “mental wellness” OR “psychological well-being” OR “emotional well-being” OR “mental health” AND “high school” OR “secondary education” OR “secondary school” AND “learners” OR “students” OR “pupils” OR “trainees.” Multimedia Appendix 1 provides a comprehensive search strategy for all the databases.

Databases vary in how subject headings are indexed. Therefore, keywords may be modified according to each database index term. Table 1 presents essential keywords for constructing the search strategy.

**Table 1.** Keywords used in the search strategy and their alternatives.

Keywords	Alternative keywords
“Psychosocial support programmes”	“Psychosocial intervention”; “emotional support”; “nursing therapy”; “psychosocial support”; “mental health program”; “psychoeducation”
“Mental well-being”	“Mental wellness”; “psychological well-being”; “emotional well-being”; “mental health”
“High school”	“Secondary education”; “secondary school”
“Learners”	“Students”; “pupils”; “trainees”

#### Searching the Reference of Identified Studies and Other Sources

The reference lists of the identified studies will be reviewed to find additional studies. This step is essential to ensure that all potential sources are considered, minimizing the likelihood of excluding relevant studies.

### Selecting the Evidence

#### Overview

Two reviewers will independently screen titles, abstracts, and full texts using EndNote and Covidence. Discrepancies will be resolved through discussion or by a third reviewer.

#### A Review of the Inclusion Criteria and Eligibility Criteria

##### Population, Concept, and Context

Studies will be chosen based on the following criteria: (1) population: primary research will be reviewed with high school learners aged 12 to 18 years. In South Africa, any establishment that provides a range of grades from grade 8 to grade 12 is considered a high school [18]; (2) concept: psychosocial support programs that promote the mental well-being of high school learners will be included. Psychosocial programs that do not focus on this will not be included, as not all interventions are tailored to this; and (3) context: this review will only include studies from LMICs to determine what psychosocial support programs are available, as they are scarce compared to high-income countries.

##### Types of Studies

Quantitative, qualitative, and mixed methods study designs will be considered for inclusion in this scoping review. The context of the current review will not be restricted to opinion papers and other similar material, as the material will not be able to accomplish the goal of the psychosocial support programs, which is to evaluate their impact on high school learners. The data provided by systematic reviews and meta-analyses is based on a pool of interventions and may not be relevant to the scoping review. The purpose of this review is to map and describe primary research evidence on the development and implementation of psychosocial support programs for high school learners’ mental health in LMICs. Including systematic reviews or meta-analyses could lead to duplication of evidence, as their findings are already synthesized from primary studies that may also be included in this review. Moreover, scoping reviews that aim to explore how interventions are designed and implemented benefit more from analyzing original study data rather than secondary syntheses. Research articles written in languages other than English may need to be translated, which could compromise the accuracy and consistency of data extraction. This may limit the scope of evidence; however, the decision is based on both practical and methodological considerations. In addition, most major databases (eg, PubMed, PsycInfo, and ScienceDirect) predominantly index English-language journals, which means non-English studies are less likely to be captured in standard systematic searches and will deviate the methodology from being consistent. Thus, they will be excluded from this study. An initial search was conducted, and it yielded thousands of articles. Only articles published between 2013 and 2023 will be included to narrow the focus. Articles outside of this time frame will be excluded.

##### Quality Assessment of Data

Contrary to systematic reviews, scoping reviews do not require a quality data assessment [19]; however, the utility of the review’s findings may be compromised if the quality of the scoping review is not assessed. Thus, depending on the review’s goal, some authors propose that this may be done at the reviewers’ discretion. Two reviewers will ensure the quality of the data is maintained.

### Gathering the Evidence

Moola et al [20] state that charting the results, sometimes called extraction of the data, provides a clear and concise summary of the findings. The charting table is updated automatically throughout the iterative process. The data will be extracted using the information highlighted below: author(s), year of publication, origin or country, study population, sample size, methodology, type of intervention, and findings.

### Analysis of Data

#### Data Extraction and Refinement

The researcher will extract data, and the supervisors will review the data; a meeting will be held to determine consensus. If consensus cannot be achieved, a third party will be asked to evaluate the articles and the content analysis approach outlined in Elo and Kyngäs [21] and Sandelowski [22] will be used for data analysis. This process will include data that will be coded independently by 2 reviewers using a predefined coding template, with the possibility to add new codes that may emerge during the analysis. Codes will then be organized into categories and further developed into themes through an iterative process of discussion and comparison both within and between studies. Any disputes will be settled by consensus or with a third party. An audit trail will be maintained to record coding decisions and theme development. The results will be presented as descriptive narratives, with tables and figures to support them.

#### Expected Results

This review will analyze psychosocial support programs for high school learners in LMICs, identifying gaps in the literature and highlighting areas for further investigation, thereby aiding in creating or adjusting such programs.

### Presentation of the Findings

Findings will be presented in tables and graphs, including the components of the PCC, which aligns with the objectives and the research question of this scoping review [19].

### Summary of the Findings

Findings will be summarized numerically (eg, study distribution by year and region) and narratively (eg, key themes). Tables and figures will present the detailed PCC.

### Ethical Considerations

Ethics approval was not required for this scoping review. The review will synthesize data from existing, publicly available literature and does not involve the collection of primary data or interaction with human participants. According to the *JBI Manual for Evidence Synthesis* [17], scoping reviews that use secondary data do not require a formal ethics approval. In addition, the review will be conducted in accordance with the principles of research integrity and reporting transparency. All sources of data will be referenced, and the review will be conducted according to the reporting framework for the PRISMA-ScR to ensure methodological rigor and transparency. Results will be shared through a peer-reviewed journal and conference presentation to contribute to the research, practice, and policy debates on school-based psychosocial support. Along with results from the study being published in a peer-reviewed journal and presented at conferences, school practitioners, policymakers, and administrators will be provided with research summaries and briefs. These deliverables will provide critical findings, evidence gaps, and potential pathways for action to inform the design and delivery of psychosocial support programs for high school students in LMICs. Other dissemination activities might involve webinars, workshops, or policy briefs to promote the practical use of the results. Patients and the public were not involved in this research's design, reporting, or dissemination plans.

## Results

This scoping review is not a funded study, and data collection was completed in May 2025. Data analysis is currently in progress, and the results are expected to be finalized and submitted for publication in February 2026.

## Discussion

### Anticipated Findings

This scoping review is anticipated to give a general account of the nature of development and delivery of psychosocial support interventions for high school learners from LMICs. Results will inform the understanding of common approaches, key components of successful interventions, and contextual factors affecting their delivery. Although the review may identify useful implications for policy and practice in the future, this review is designed to map the evidence, rather than evaluate effectiveness or provide recommendations for policy. The results are expected to inform further investigation and evaluation studies to enable evidence-based psychosocial interventions to be adapted and scaled up and enhance support for mental health in the school environment.

The review will pinpoint the essential components of successful psychological treatments, point out weaknesses in current initiatives, and investigate the external circumstances impacting their execution and efficacy.

The discussion will examine, through an analysis of the research on school-based interventions, how these programs support mental health for high school learners in environments with limited resources, highlighting their capacity to improve coping mechanisms, resilience, and general well-being.

Additionally, the research findings will offer suggestions to legislators, educators, and mental health professionals, assisting them in creating and executing more focused and long-lasting psychosocial treatments. Important knowledge gaps regarding mental health support in LMICs will be filled by identifying areas that need more exploration. Through this review, the study will contribute to the broader discussion about fair access to mental health services in educational settings, ultimately promoting more robust integration of psychosocial support in school systems. The review protocol adheres to the JBI scoping review framework, ensuring systematic methodology, reliability, and validity of the findings.

A thorough search across 5 databases, gray literature, and Google Scholar will be conducted, aligning with defined eligibility criteria. Additionally, the screening process and data extraction involve 2 independent reviewers to enhance transparency, reliability, and minimize bias. It is important to note that the inclusion criteria limit the articles to those written in English and published after 2024. Despite these restrictions, the results from the scoping review will provide insight into the factors that both support and hinder program efficacy, such as institutional support, financial limitations, and cultural considerations.

### Strengths and Potential Limitations of This Study

While several scoping review studies have been conducted on the mental health of high school learners, little is known about the development and implementation of psychosocial support programs for this population in LMICs. As a result, this scoping review will provide insight into the program design, implementation procedures, and contextual elements that affect the provision of psychosocial support for the mental health of high school learners in LMIC settings. Two independent reviewers will choose the studies and extract the data to guarantee accuracy and interrater reliability. Only studies published in English will be included in the screening process, which may result in omitting important information in other languages. The evidence will only relate to psychosocial support programs developed and implemented in high schools located in LMICs.

There will be several potential limitations to this scoping review. Limiting the inclusion criteria to English-language studies may result in language bias. There may also be selection bias in the choice of databases and publication bias, as only published sources will be considered. To address these concerns, we will screen gray literature and reference lists of included studies for additional eligible publications.

### Conclusion

This review will synthesize evidence on psychosocial support programs in LMICs and guide the development of targeted interventions to address the mental health needs of high school learners.

## Supplementary material

10.2196/78035 Multimedia Appendix 1 Database search strategy.

10.2196/78035 Checklist 1 PRISMA checklist.

## Abbreviations

JBI: Joanna Briggs Institute
LMIC: low- to middle-income country
PCC: population, concept, and context
PRISMA-ScR: Preferred Reporting Items for Systematic Reviews and Meta-Analyses Extension for Scoping Reviews
