# Supplementary material for: Psychosocial Support Programs for the Mental Well-Being of High School Learners From Low- to Middle-Income Countries: Protocol for a Scoping Review
Source: JMIR Res Protoc. 2025 Dec 30;14:e78035. doi: 10.2196/78035 (PMC12753099; doi:10.2196/78035)
Supplement: Multimedia Appendix 1 [file resprot-v14-e78035-s001.doc]

| **Database** | **Date of search** | **Search Criteria** |
| --- | --- | --- |
| **ERIC** | 19.01.2024 | (Psychosocial support or psychological intervention or emotional support) AND (mental well-being or mental health or psychological wellbeing) AND (high school or secondary education) AND (learners or students or pupils) AND low-middle income countries. |
| **Medline** | 19.01.2024 | Psychosocial support OR intervention OR emotional support AND mental well-being OR mental health AND High school OR Secondary education AND learners OR students AND low-middle income countries  Filters 2013-2023; full text; English; mental health; high school students |
| **Psych Info** | 19.01.2024 | Psychosocial support OR intervention OR emotional support AND mental well-being OR mental health AND High school OR Secondary education AND learners OR students AND low-middle income countries  Filters 2013-2023; full text; English; mental health; high school students |
| **ScienceDirect** | 19.01.2024 | Psychosocial support AND mental well-being AND high school AND learners AND low- middle-income countries Year: 2013-2023 |
| **Pubmed** | 19.01.2024 | ((((((((((Psychosocial support) OR (psychosocial intervention)) OR (emotional support)) AND (mental wellbeing)) OR (mental health)) OR (High school))) OR (secondary education)) AND (learners)) OR (students)) AND (low-middle-income countries) Filters: 2013-2023;  full text; clinical study; English; Humans |
